# Tracking Down a Cause for Hypospadias: Placental Malfunction May Contribute

**Published:** 2008-08

**Authors:** Angela Spivey

Hypospadias is a male birth defect in which the opening of the urethra is located on the underside of the penis instead of the tip. Although the defect is common and increasing in prevalence (2–8 cases per 1,000 live births in Western countries), the causes of most cases are unknown. Some studies suggest that reduced levels of the placental hormone human chorionic gonadotropin (hCG) may play a role; others suggest associations between hypospadias and conditions such as low birth weight, preterm birth, and preeclampsia that could be caused by malfunction of the placenta and subsequent abnormalities in hormone regulation and nutrition provided to the fetus (a condition known as placental insufficiency). A new study now presents additional evidence that hypospadias has its origins in malfunction of the placenta **[*EHP* 116:1071–1076; Akre et al.]**.

Data on 292 cases of hypospadias and 427 controls were collected as part of a joint Danish–Swedish study of both hypospadias and cryptorchidism (undescended testes). In Sweden, hypospadias cases were recruited at a pediatric surgery clinic, and data were collected via self-administered questionnaires. In Denmark, cases were from the Danish National Birth Cohort, a population-based cohort of women and children. Mothers were interviewed while pregnant and twice after delivery. Matched controls were born within at least 6 months of each case and within the same county, and were randomly selected from national birth and population registries.

The investigators found several conditions independently associated with increased hypospadias risk, most of which they say could be explained by impaired production of hormones by the placenta. Mothers without first-trimester nausea were twice as likely to bear sons with hypospadias, as were mothers who had a prepregnancy body mass index of 30 or greater. These findings support the theory that placental insufficiency contributes to hypospadias. Nausea is believed to be caused by an early surge of pregnancy hormones, and the absence of first-trimester nausea is associated with low hCG levels. A previous study showed that obese women have lower levels of a family of proteins called plasminogen activator inhibitors, some of which are derived from the placenta.

The team also determined that a maternal diet lacking both fish and meat was associated with more than a fourfold increased risk of hypospadias in baby boys. This finding complements a 2000 study by other authors that showed a strong positive association between maternal vegetarian diet and hypospadias in offspring. The authors of the current study conclude that exclusion of animal proteins could increase the risk of a transient deficiency of some nutrient that’s essential for formation of the organs or the placenta. Another explanation is that some protein sources in vegetarians’ diets (such as soybeans) contain compounds with hormonal effects that may affect the development of the urogenital organs in humans.

